# Development and psychometric evaluation of the Khaini Smokeless Tobacco Dependence Scale

**DOI:** 10.18332/tid/160073

**Published:** 2023-03-15

**Authors:** Vaibhav P. Thawal, Christine Paul, Erin Nolan, Flora Tzelepis

**Affiliations:** 1 School of Medicine and Public Health, University of Newcastle, Newcastle, Australia; 2 Hunter Medical Research Institute, Newcastle, Australia; 3 Priority Research Centre for Health Behavior, University of Newcastle, Newcastle, Australia; 4 Priority Research Centre for Cancer Research, Innovation and Translation, University of Newcastle, Newcastle, Australia; 5 Hunter New England Population Health, Hunter New England Local Health District, Newcastle, Australia

**Keywords:** dependence, psychometric properties, scale development, khaini, smokeless tobacco

## Abstract

**INTRODUCTION:**

Khaini is a smokeless tobacco (SLT) product commonly used in the South-Asian region. It is the most common smokeless tobacco product used in India, having a prevalence of 11.2% and is used by 104.1 million adults. No scales exist to assess khaini dependence. Existing scales available to assess dependence on smokeless tobacco products are not ideal as these are adapted from cigarette dependence scales and developed for western populations. This study aimed to develop a khaini dependence scale and assess its reliability and validity.

**METHODS:**

Recommended methods for scale development were followed for item development, scale development and scale evaluation. Scale development was guided by a theoretical framework, a review of existing scales and in-depth interviews with 21 khaini users recruited from a tertiary care hospital in Mumbai, India. The process involved the identification of domains for dependence and the development of an item pool. Cognitive interviews and pre-testing were conducted with 20 khaini users to assess content validity. A cross-sectional survey with 323 khaini users was conducted, and Exploratory Factor Analysis (EFA) was used to determine the factor structure of the draft scale. The content validity, criterion validity (by cross-referencing with the cotinine level of users), convergent validity and internal consistency of the new scale were assessed.

**RESULTS:**

The final version of the Khaini SLT Dependence Scale (KSLTDS) had 20 items. EFA indicated an acceptable goodness of fit for a three-factor structure with physical, psychological and sociocultural-behavioral sub-scales. It showed evidence of acceptable criterion validity with cotinine (ρ=0.43, p=0.0002), convergent validity with FTND-ST (ρ=0.51, p<0.0001) and frequency of khaini use (ρ=0.38, p<0.0001). The sub-scales (α=0.87–0.90) showed acceptable internal consistency.

**CONCLUSIONS:**

The psychometric evaluation of the KSLTDS showed preliminary validity and reliability for assessing dependence on khaini, and therefore, it is appropriate for clinical and research purposes. Re-validation studies are required with various khaini user populations.

## INTRODUCTION

Smokeless tobacco (SLT) use is a global problem, with around 313 million users worldwide and 256 million (82%) living in South-East Asia^1^. Low-and-middle-income countries bear the highest burden of SLT-related diseases^2^. Consumption of SLT may cause various health effects, including cancers, cardiovascular, and pregnancy-related disorders^2^. SLT products are used worldwide with unique usage patterns, product characteristics, sociocultural factors and beliefs^3^.

SLT products have high levels of nicotine, making them highly addictive^3^. SLT users have also been shown to have high cotinine levels (380 ng/mL) compared to combustible tobacco users^3^. With the variations in the methods (sucking, chewing), there are variations in pH and higher nicotine absorption resulting in higher addiction from SLT products^3^. The pH levels vary across products (9.47 to 5.24, highest for khaini use and lowest for loose tobacco)^3^. Blood nicotine levels drop slowly among SLT users, and the nicotine absorption continues even after tobacco consumption which is different in cigarette smokers, where the level drops off rapidly after smoking^3^. In developed countries, dip, snuff and snus are the most commonly known form of SLT; however, in South-Asian countries, SLT comes in many forms^3^. Common SLT products consumed in India, Nepal, Sri Lanka, Bangladesh, Pakistan, Myanmar, and Bhutan are betel-quid, khaini, gutkha, gul, gudakhu, zarda, and dry-snuff ^3^.

India has a high prevalence of SLT, with 21.4% of all adults (199 million) using some form of SLT^4^. Khaini is an oral SLT product used across South-Asian countries, mainly in India, Bangladesh, Nepal and Bhutan^3^. It is the most common product used in India, with 11.2% (104.1 million) of adults aged ≥15 years using khaini^4^. Khaini is a product made by rubbing a pinch of sun-dried tobacco coarsely cut tobacco leaves together with slightly moistened slaked lime (calcium hydroxide) paste^3^. This mixture is held between the gums and the buccal mucosa for 5–20 minutes; and is used between 3–30 times a day^3^. Khaini is identified as a hazardous product as it has a damaging effect on the chromosomes and tumors suppressor genes resulting in oral cancers^3^. The risk of hypopharyngeal cancer is higher in khaini users compared to never users^3^.

It is well established that dependence is a major barrier to tobacco cessation^5^. Scales assessing dependence on tobacco products are important components of cessation programs as they help to measure the severity of dependence and inform treatment planning^5^. There are sixteen scales developed to assess dependence on SLT, of which fourteen^6-14^ are general, and two are product specific, Betel Quid Dependence Scale (BQDS) and Betel Quid Dependence Instrument (BQDI) ^15,16^. Of the 16 scales, 13 were developed in the USA^6-13^, two in Taiwan^15,16^ and one in Sweden^14^. None of the scales was developed in the South-Asian region despite South Asia having the highest prevalence of SLT use^1^.

The only study evaluating existing SLT dependence scales, Oklahoma Scale for Smokeless Tobacco (OSSTD), Tobacco Dependence Screener (TDS) and Fagerström Test for Nicotine Dependence-Smokeless Tobacco (FTND-ST) in South-Asia (Bangladesh) found the OSSTD lacked construct validity due to differences in the tobacco products, usage behaviors and sociocultural factors in Bangladesh and highlighted the need to develop new SLT scales relevant for the South-Asian population^17^. Most of the early tobacco dependence scales were developed for cigarettes^18^. Using traditional scales with simple adaptations to other products may not be ideal for assessing SLT dependence. Literature on scale development emphasizes the need for extensive interaction with the relevant populations^19^. Although the FTND-ST scale showed good reliability and validity in the same evaluation study, the unidimensional structure assessing only physical dependence limits understanding of the multidimensional aspects of dependence on SLT use^17^. The FTND-ST includes an item on time to first use (TTFU), which makes the scale less relevant for assessing dependence on SLT products in the Indian context^20^. This is because there is variation in TTFU across smokeless tobacco products and smokeless tobacco users^20^. For example, mishiri a SLT product in India, is used as a dentifrice for cleaning teeth and is used within 5 minutes of waking. On the other hand khaini, gutkha or betel quid are first used later in the day^20^.

Tobacco dependence is not limited to physiological and psychological dependence on the product but is also related to product specific behaviors (how it is acquired, prepared, and consumed) with its unique cultural context^21^. Thus, only using biological measures (cotinine analysis) that assess physical dependence does not capture all aspects of dependence^22^. SLT products are acquired, prepared, and consumed in various ways that shape the tobacco use behavior:

1. Acquired/prepared – custom-made by tobacco vendors (mawa, betel-quid) that needs immediate consumption and cannot be stored; self-made (khaini can be prepared as needed); or available in packaged forms for single use (e.g. gutkha).
2. Consumption – ‘when it is used’ (in the morning, e.g. mishiri); ‘where it is used’ only at home as it needs a mouth wash after use (e.g. mishiri) or anywhere at home/workplace (khaini or gutkha); needs continuous chewing and spitting the saliva/product (gutkha, mawa, betel quid); and can be sucked for longer duration without the need to continuously spit (e.g. khaini)^3^.

These factors shape how an individual uses a specific SLT product in a day and affect indicators of dependence such as the frequency and patterns of use, triggers, cues for use, and sociocultural environment that promotes use^3^. Thus, existing SLT dependence scales developed to assess dependence on SLT products in developed countries may not be appropriate for SLT users in South Asia and India.

There is also a need to assess social and cultural norms that may affect an individual’s tobacco use; for example, acceptance of use in specific contexts, religious affiliations, and stigmatization in certain populations such as women and people with low socioeconomic status^21^. For both clinical and research purposes, capturing all factors that contribute to tobacco/nicotine dependence will likely require assessment instruments that are product specific^18^. Literature on different tobacco products emphasizes the variations in pharmacokinetic and pharmacodynamics effects as well as the sensory and behavioral involvements of various tobacco products. Tobacco products have unique behaviors and stimuli and using traditional scales developed for assessing dependence may not be appropriate^18^. To capture the dependence produced by different tobacco products there is a need for product-specific instruments^18^. Assessing tobacco dependence requires a product-specific scale that includes an assessment of pharmacology, product characteristics, accompanying behaviors and stimuli^23^. Two product-specific dependence scales, BQDS and BQDI, for assessment of smokeless tobacco product betel-quid have been developed to date^15,16^. Depending on the type of SLT product, nicotine content may vary, and therefore, measurement of nicotine and its metabolites among SLT users is important to understand the addictive potential of SLT products^18^. Considering the need for product-specific scales^18^ which are developed in the relevant social and cultural context, there is an emerging need for a scale to assess dependence on SLT products consumed in India. These tools will help in the accurate diagnosis of dependence and help in planning treatment strategies^5^.

Although khaini is one of the most commonly used SLT products in India, no standardized instrument is available to assess dependence on this product. The study aimed to develop a product-specific scale to assess dependence on khaini, its content, construct, criterion, convergent validity and internal consistency.

## METHODS

**Figure 1:**
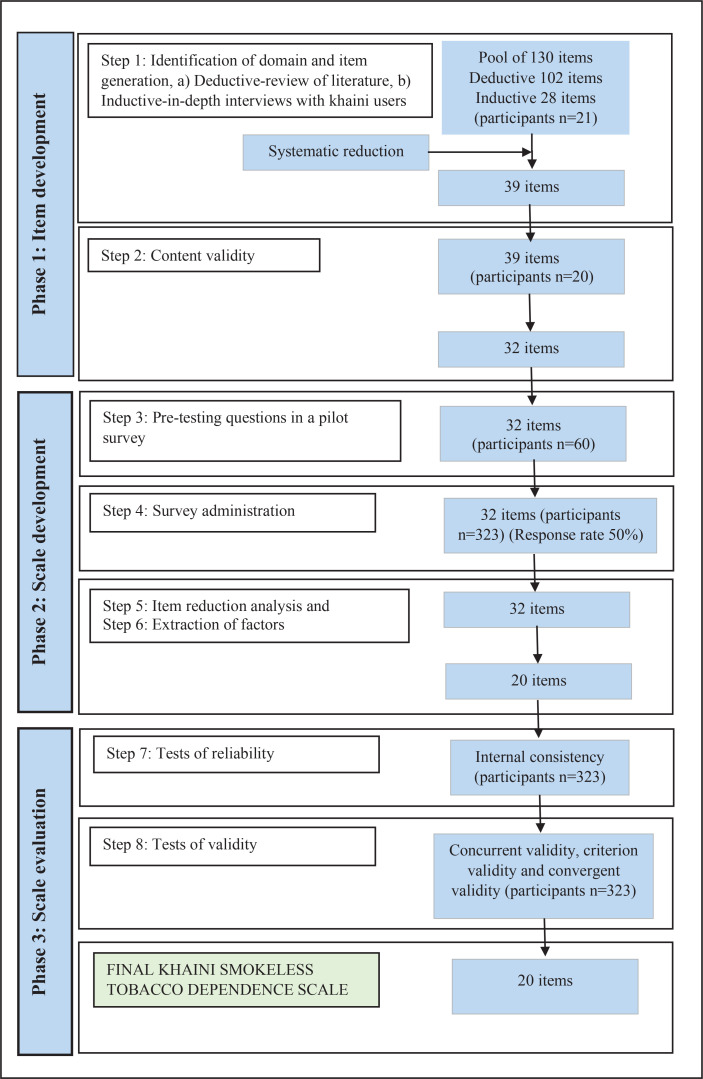
Scale development process

**Figure 2:**
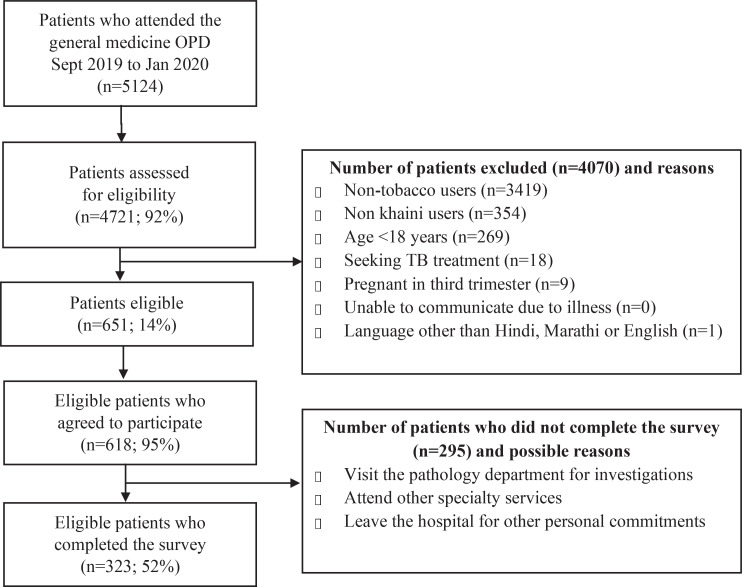
Recruitment flowchart

The scale was developed using a systematic approach and evaluated using an eight-step process over three phases^19^ (Figure 1). Between June 2019 and January 2020, the study was carried out in a 300-bed multi-specialty hospital with an average of 1200 to 1300 patients a day visiting the Outpatient Department (OPD) catering to a population of around 400000. Please refer to Figure 2 for details on the recruitment process.

### Phase 1: Item development

*Step 1: Identification of domains and item generation*

The study was guided by a theoretical framework drawing on a list of domains for initial conceptualization of the construct ‘dependence’^24,25^. A literature review was also conducted to identify domains of dependence (Supplementary file). The item development followed recommended practice involving both deductive and inductive methods^19^. Items related to domains of dependence were developed via a literature review of smokeless tobacco dependence scales and from in-depth interviews with khaini users. In-depth interviews were conducted with 21 exclusive khaini users by the first author (male) and a research assistant (female) with khaini users to understand the patterns, motives/reasons, expectations from use, and the social/cultural context of khaini use. A maximum variation sampling approach and quotas were used to ensure diversity in gender, age, education level, occupation, and frequency of khaini use among the participants. The theoretical framework^24,25^ informed development of the interview guide, which examined: 1) patterns and the current reasons for using khaini; 2) reasons for initiation and changes in reasons over the period; 3) change in frequency of use in different situations; 4) experiences of past abstinence and perceptions about future abstinence; 5) role of tobacco use in life; and 6) cultural and social factors/reasons for using khaini. The interviews were conducted face-to-face in a quiet, private room in the OPD and were audio recorded and transcribed. Themes were derived from the interview transcripts by grouping and classifying codes according to relevant domains. Participants’ terminology for referring to khaini use was also extracted to guide the wording of scale items.

Scale construction: A total of 102 potential items related to the identified domains of dependence were selected from the identified SLT scales. These items were mapped to the themes and codes from the in-depth interviews and 28 additional items were created by the authors based on the interviews generating a pool of 130 items. The authors then refined the total pool of items to produce new items, using frequent comparisons to the literature and qualitative work. In the initial step, the authors reviewed the entire pool of items to assess relevance and redundancy, and after removing items with similar content and expressions, 62 items were retained. Consensus decisions on whether an item was retained or not were made through discussion within the research team. In the following step, assessment of the content of items was conducted based on relevance, importance, the occurrence of response from khaini users and significance attached to it as a valid indicator of dependence on khaini use. During this process, 23 items were dropped, retaining 39-items for further evaluation. This process produced a draft Khaini Smokeless Tobacco Dependence Scale (KSLTDS) containing 39 items. A 4-point Likert scale (1 = ‘strongly disagree’ to 4 = ‘strongly agree’) was used for all items (except the first three items) for ease of understanding. Negative items were reversed scored during the analysis. The first three items ‘item 1- frequency of khaini use’; ‘item 2- average dipping time in minutes’, and ‘item 3- first use on waking up’ were open-ended items. The draft scale was translated in Hindi and Marathi languages for conducting cognitive interviews with khaini users. Please refer to the Supplementary file for details about the translation process.

*Step 2: Content validity*

Cognitive interviews were conducted with 20 khaini users (10 Marathi, 10 Hindi) to explore their thoughts about each scale item and response. A maximum variation sampling approach was used to select participants. During the interview, participants were asked to read and complete the draft KSLTDS and to explain their understanding of each item and the corresponding response options. Items were also discussed in terms of their acceptability, clarity, redundancy and importance. After cognitive interviews, fourteen items were reworded and further simplified as per the feedback received from the participants. Seven items were deleted as they were difficult to understand and confusing to the participants.

### Phase 2: Scale development

*Step 3: Pre-testing questions*

After conducting cognitive interviews with participants, the draft KSLTDS was pre-tested in a pilot study with 60 exclusive khaini users to assess comprehensibility of language and phrasing, ease of administration and responding, and any other implementation issues. The pre-testing confirmed that the language, phrasing and wording of items were acceptable and that no further changes were required prior to validation.

*Step 4: Survey administration*

A cross-sectional study was carried out between September 2019 and January 2020. Patients visiting the general OPD were approached by the research assistant in the waiting area and were invited to participate in the survey. Participants who were aged ≥18 years, exclusive khaini users (used in past 30 days on a daily/less-than-daily), spoke English, Hindi or Marathi were eligible to take part in the survey. Excluded participants were those seeking tuberculosis treatment (for safety of the research team), pregnant women (third trimester), and patients with serious illness. Eligible participants were referred to a dedicated OPD where they provided written consent and completed the questionnaire in the presence of an investigator. For participants who had literacy levels which limited their ability to complete the survey on their own, the questions and response options were read aloud to the participants. The required sample size for the survey was estimated using the recommended ratio of 10 participants per scale item^19^. To test the psychometric properties of the KSLTDS, a cross-sectional study (n=323) was carried out to refine item composition and examine the validity and reliability of the 32-item KSLTDS. Please refer to Figure 2 for details on recruitment process. The data from the cross-sectional survey was used for extraction of factors and assessing reliability and validity of the KSLTDS.

*Step 5: Item reduction analysis and Step 6: Extraction of factors*

Inter-item correlation and item-total correlation were estimated with <0.30 the accepted threshold for retention of items^19^. To determine the potential number of factors present within the draft scale, three criteria were considered: 1) Scree plot of eigenvalues for each factor number, with eigenvalues >1 and ‘elbow’ angle of improvement indicative of optimal factors; 2) proportion of variance explained ≥0.05; and 3) number of factors that had ≥3 items with a rotated factor loading ≥ |0.4| ( ≥0.4 or ≤ -0.4)^19,26^. Further factors with at least 3 items with factor loading ≥0.40 were retained^19^. The maximum likelihood method was used to extract factors. The prior communality estimate for each variable was calculated using its squared multiple correlation with all other variables. The oblique ProMax rotation was used as it makes no assumptions about the correlation between factors. We focused on an iterative exploratory factor analysis (EFA) to help understand the factor structure of the KSLTDS and for item reduction. To decide which items should be removed, the following observations were considered: items with a loading of <0.4 on any factor, items with low item-total correlation (<0.30) to the total questionnaire, items whose removal improved internal consistency of the factor, items whose removal reduced redundancy, and items that were clinically less important and whose deletion would not affect the entire scale^19^. Items that were considered important to the fundamental domain of dependence were retained. Model fit was evaluated using the standardized root mean square residual (SRMR) with acceptable thresholds for satisfactory model fit ≤0.08^19^.

### Phase 3: Scale evaluation

*Step 7: Reliability and Step 8: Validity*

All analyses were performed using SAS 9.4. EFA was performed and the resulting factors were assessed for validity and internal consistency^19^. To verify the internal consistency of the KSLTDS, Cronbach’s alpha coefficient of the subscales was calculated, with an acceptable value of α between 0.70 and 0.95^19^. Convergent validity was determined by measuring the Pearson correlation between the frequency of khaini use, KSLTDS factors, KSLTDS total score and the total score of the Fagerström Test for Nicotine Dependence - Smokeless Tobacco (FTND-ST)^7^. The FTND-ST is a six-item continuous scale with scores ranging from 0 to 10 with higher score indicating higher dependence on SLT. Salivary cotinine, which is considered a gold standard, was used to assess criterion validity^6^. NicAlert, a widely used semi-quantitative rapid assessment kit was used to measure saliva cotinine levels. Refer to the Supplementary file for details on NicAlert. Criterion validity was determined by using Pearson correlation analysis between the factors, total score and the cotinine score.

## RESULTS

### Participant characteristics

The demographic characteristics and tobacco use characteristics of participants who completed the in-depth interviews, cognitive interviews, and survey are presented in Table 1.

**Table 1:** Demographic characteristics of participants recruited in the three phases of the study, India, 2019–2020

*Characteristics*	*In-depth interviews (N=21) Phase 1*		*Cognitive interviews (N=20) Phase 2*		*Survey (N=323) Phase 3*	
	*n*	*%*	*n*	*%*	*n*	*%*
**Gender**						
Male	12	57.14	10	50.00	205	63.40
Female	9	42.86	10	50.00	118	36.60
**Age** (years)						
Mean (SD)	39.66 (10.61)		40.65 (8.65)		34.45 (12.37)	
Median	39		40		32	
**Education level**						
No formal schooling	4	19.04	5	25.00	41	12.73
Primary school	3	14.29	8	40.00	88	27.33
Secondary and higher	14	66.67	7	35.00	194	59.94
**Occupation**						
Government/nongovernment employee	9	42.86	5	25.00	136	41.93
Self-employed	4	19.05	8	40.00	66	20.50
Student	2	9.52	0	0.00	22	6.83
Homemaker	5	23.81	6	30.00	76	23.60
Retired or unemployed	1	4.76	1	5.00	23	7.14
**Marital status**						
Single	3	14.29	2	10.00	80	24.80
Married	17	80.95	18	90.00	224	69.30
Divorced	0	0	0	0.00	0	0.00
Separated	0	0	0	0.00	0	0.00
Widowed	1	4.76	0	0.00	19	5.90

SD: standard deviation.

### Initial item analysis

Item discrimination was assessed via corrected item-total correlations. Corrected item-total correlations (using the entire scale) ranged from r = -0.64 to 0.82 (with six items having low correlation <0.30). The remaining items met the criteria for adequate item discrimination (i.e. r ≥0.30) (Supplementary file Table 1). In total, 12 items were dropped: items 2, 3, 7, 15, 21 and 31 had a low correlation and hence were dropped, and items 1, 17, 19, 24, 26 and 32 were removed based on low clinical importance to reduce redundancy.

### Content validity

The content validity of the tool was ascertained at the beginning of the study, administering the scale to a group of khaini users. Items that needed to be clarified for the participants were deleted. The process also identified appropriate keywords and language that were understandable by the targeted population and relevant for khaini use in the Indian context. This approach strengthened the content validity of the scale.

### Construct validity

EFA was used to identify the underlying factor structure. The final EFA indicated a three-factor structure. Factor 1, ‘physical dependence’, included five items (4, 5, 11, 23 and 25) associated with the physical dependence, i.e. the cravings and withdrawal symptoms related to khaini use. Factor 2, ‘psychological dependence’, contained seven items (6, 8, 12, 13, 14, 16 and 22) associated with the psychological dependence, i.e. the cues, urges, and the positive/negative associations related to khaini use. Factor 3, ‘Sociocultural and behavioral dependence’ consisted of six items (9, 18, 20, 27, 28 and 29) associated with the sociocultural and behavioral factors related to khaini use. Item loadings for this model are presented in Table 2. There was no cross-loading onto other factors. The number of factors retained was based on the theoretical interpretability of the factor loading pattern as a criterion for selecting a model to achieve a multidimensional measure. The SRMR = 0.04 indicated that the three-factor model had adequate goodness of fit. Within subscales, the item-total correlations ranged from r = 0.71 to 0.81 for the ‘physical dependence’, r = 0.36 to 0.84 for the psychological dependence, and r =0.43 to 0.83 for the ‘sociocultural and behavioral’ subscale (Table 3). Although items 10 and 30 did not load onto any of the factors, they were retained in the final scale because of their clinical importance and importance to the fundamental domain of dependence. The final KSLTDS contained 20 items (Table 4).

**Table 2:** Factor loadings for the 3-factor structure of the final KSLTDS scale, Phase 3 scale evaluation (Step 4: Survey administration), India, 2019–2020 (N=323)

*No.*	*Questionnaire item*	*Pattern*		
		*Factor 1*	*Factor 2*	*Factor 3*
1	Item 4. I experience cravings if I don’t use for 30 minutes	0.72	-0.20	0.23
2	Item 5. My cravings get stronger if I don’t use	0.80	0.10	-0.02
3	Item 6. I find myself using khaini routinely without cravings	-0.00	0.67	0.24
4	Item 8. I use khaini every time I go to the washroom	0.10	0.64	-0.21
5	Item 9. My khaini use is a routine habit similar to eating	0.17	0.32	0.46
6	Item 10. I use khaini when I have to focus on a task	0.31	0.21	0.08
7	Item 11. I sometimes wake up at night to use khaini	0.72	-0.04	0.09
8	Item 12. I feel bored if I don’t use khaini for some time	-0.18	0.55	-0.08
9	Item 13. If I don’t use khaini I feel I am missing an important daily activity	-0.03	0.82	0.15
10	Item 14. I use khaini more when I am sad, tense, stressed or worried	0.07	0.81	0.01
11	Item 16. I feel anxious when I don’t have khaini available with me	0.12	0.72	-0.02
12	Item 18. I have gradually increased the amount of khaini use from the first time I started using it	0.08	0.26	0.47
13	Item 20. I use khaini after meals or tea	0.04	-0.23	0.63
14	Item 22. I get drowsy if I don’t use khaini for some time	0.32	0.48	0.15
15	Item 23. I would continue using khaini even if I have cancer sores, mouth ulcers or loose teeth	0.97	-0.04	-0.14
16	Item 25. It would be difficult for me to quit my khaini use completely	0.60	0.13	0.23
17	Item 27. I am aware of the quantity of khaini and plan to buy more so I won’t run out	-0.06	0.05	0.84
18	Item 28. I use khaini in front of my friends	0.16	0.16	0.66
19	Item 29. I use khaini in front of my family	0.25	0.05	0.44
20	Item 30. I will share khaini with others so they will share with me when I need it	0.33	0.24	0.17

Factor 1: Physical dependence. Factor 2: Psychological dependence. Factor 3: Sociocultural and behavioral dependence.

**Table 3:** Internal consistency of subscales of the KSLTDS, Phase 3 scale evaluation (Step 4: Survey administration), India, 2019–2020 (N=323)

*No.*	*Items*	*Correlation ρ*	*Cronbach’s alpha*
**Internal consistency of the factor: ‘Physical dependence’**			
	Overall		0.90
1	Item 4. I experience strong cravings for khaini when I don’t use for 30 minutes	0.74	0.88
2	Item 5. My cravings get stronger if I don’t use khaini	0.78	0.87
3	Item 11. I sometimes wake up at night to use khaini	0.71	0.89
4	Item 23. I would continue using khaini even if I have cancer sores, mouth ulcers or loose teeth	0.81	0.87
5	Item 25. It would be difficult for me to quit my khaini use completely	0.74	0.88
**Internal consistency of the factor: ‘Psychological dependence’**			
	Overall		0.88
1	Item 6. I find myself using khaini routinely without cravings	0.75	0.86
2	Item 8. I use khaini every time I go to the washroom	0.53	0.88
3	Item 12. I feel bored if I don’t use khaini for some time	0.36	0.91
4	Item 13. If I don’t use khaini I feel I am missing an important daily activity	0.84	0.84
5	Item 14. I use khaini more when I am sad, tense, stressed or worried	0.79	0.85
6	Item 16. I feel anxious when I don’t have khaini available with me	0.74	0.86
7	Item 22. I get drowsy if I don’t use khaini for some time	0.74	0.86
**Internal consistency of the factor: ‘Sociocultural and behavioral dependence’**			
	Overall		0.87
1	Item 9. My khaini use is a routine habit similar to eating or other daily activities	0.76	0.84
2	Item 18. I have gradually increased the amount of khaini I use form the first time I started using it	0.66	0.86
3	Item 20. I use khaini after having meals or tea	0.43	0.89
4	Item 27. I am aware of the quantity of khaini left in my pouch/can and plan to buy more so I won’t run out	0.77	0.84
5	Item 28. I use khaini in front of my friends	0.83	0.82
6	Item 29. I use khaini in front of my family	0.60	0.87

ρ: correlation coefficient.

**Table 4:** Final Khaini Smokeless Tobacco Dependence Scale (KSLTDS)

*No.*	*Items of KSLTDS*
1	I experience strong cravings for khaini when I don't use it for more than 30 minutes
2	My cravings to use khaini get stronger if I don’t use khaini
3	I find myself using khaini routinely without cravings
4	I use khaini every time I go to the washroom
5	My khaini use is a routine habit similar to eating or other daily activities
6	I use khaini when I have to focus on a task (before or during a task)
7	I sometimes wake up at night to use khaini
8	I feel bored if I don’t use khaini for some time
9	If I don’t use khaini I feel I am missing an important daily activity
10	I use khaini more when I am sad, tense, stressed or worried
11	I feel anxious when I don’t have khaini available with me
12	I have gradually increased the amount of khaini I use from the first time I started using it
13	I use khaini after having meals or tea
14	I get drowsy if I don’t use khaini for some time
15	I would continue using khaini even if I have cancer sores, mouth ulcers or loose teeth
16	It would be difficult for me to quit my khaini use completely
17	I am aware of the quantity of khaini left in the pouch/can and plan to buy more so I won’t run out
18	I use khaini in front of my friends
19	I use khaini in front of my family
20	I will share khaini with others so they will share with me when I need it
	**Response options and score for each item**
	a) Strongly disagree (1)
	b) Disagree (2)
	c) Agree (3)
	d) Strongly agree (4)

### Internal consistency

Cronbach’s alpha for the ‘physical dependence subscale’ was α=0.90, for the psychological dependence scale was α=0.88, and for the ‘sociocultural and behavioral scale’ was α=0.87, indicating acceptable internal consistency (Table 3).

### Convergent validity

For assessing convergent validity, the Pearson correlation test was used, and KSLTDS total score was moderately correlated to the FTND-ST total score (ρ=0.51, p<0.0001). Of the three factors, ‘Sociocultural and behavioral dependence’ had the highest correlation (ρ=0.50, p<0.0001) with FTND-ST total score. The KSLTDS total score was positively correlated with the frequency of khaini use (ρ=0.38, p<0.0001) (Table 5). This indicated that the convergent validity of the KSLTDS was met, as the total score aligned with other tests measuring similar features.

**Table 5:** a) Factors scores, total score, FTND-ST and cotinine scores, b) Correlation of KSLTDS factors and total score with FTND-ST total score, and c) Criterion validity of factor groups and KSLTDS total score and cotinine test level, Phase 3 scale evaluation (Step 4: Survey administration), India, 2019–2020 (N=323)

*Score*	*Statistics*	*Measure (N=323)*	*Correlation ρ to FTND-ST*	*p*	*Correlation ρ to cotinine*	*p*	*Correlation ρ to frequency of use*	*p*
**Psychological dependence**	Mean (SD)	19.14 (4.79)	0.46	<0.0001	0.26	0.0295	NA	NA
	Median	20.00						
	(Range)	(7.00–28.00)						
**Physical dependence**	Mean (SD)	11.85 (3.40)	0.47	<0.0001	0.54	<0.0001	NA	NA
	Median	11.00						
	(Range)	(5.00–20.00)						
**Sociocultural and behavioral dependence**	Mean (SD)	16.48 (3.90)	0.50	<0.0001	0.42	0.0003	NA	NA
	Median	18.00						
	(Range)	(6.00–24.00)						
**KSLTDS total score**	Mean (SD)	52.87 (11.81)	0.51	<0.0001	0.43	0.0002	0.38	<0.0001
	Median	56.00						
	(Range)	(26.00–77.00)						
**Fagerström test**	Mean (SD)	4.99 (1.31)	1.00	-	0.36	0.0021	0.28	<0.0001
	Median	5.00						
	(Range)	(2.00–9.00)						
**Cotinine test level (n=72)**	Mean (SD)	3.82 (1.41)	NA	NA	1.00	-	0.73	<0.0001
	Median	4.00						
	(Range)	(1.00–6.00)						

KSLTDS: Khaini Smokeless Tobacco Dependence Scale. FTND-ST: Fagerström test for nicotine dependence for SLT. SD: standard deviation. NA: not applicable. ρ: correlation coefficient. p<0.05.

### Criterion validity

For assessing criterion validity, the Pearson correlation test was used, and all factors and the total score of KSLTDS were significantly correlated with the cotinine levels (Table 5).

## DISCUSSION

To our knowledge, the KSLTDS is the first scale developed to assess dependence on SLT products in South Asia. The EFA revealed a three-factor structure comprising ‘Physical dependence’, ‘Psychological dependence’ and ‘Sociocultural and behavioral dependence’ subscales. The KSLTDS showed evidence of acceptable content, construct, criterion, convergent validity and internal consistency.

The factor ‘Physical dependence’ is similar to the Fagerström scales^6,7,9^ and other SLT scales^8,11,14^. The second factor, ‘Psychological dependence’ is also similar to existing SLT scales^10-12^. The third factor, ‘Sociocultural and behavioral dependence’ represents the behavioral factors similar to the GN-STBQ^11^. In the psychological dependence sub-scale, the word ‘drowsy’ in the item ‘*get drowsy if I don’t use khaini*’ has psychological as well as physical connotations in ‘Marathi’ or ‘Hindi’ which are broader than the English meaning. Secondly, the item on ‘*gradual increase in the khaini use*’ as part of the ‘Sociocultural and behavioral dependence’ sub-scale may initially appear to relate to physical tolerance. In this context it measured the gradual increase of khaini use over the period because of its acceptance in family and social groups, as reported by khaini users in the cognitive interviews.

The KSLTDS encompasses and adds to the constructs represented in existing SLT scales. In particular, the items related to social acceptance of khaini use in front of family and friends, use of khaini every time one goes to the washroom, and sharing khaini with others to reciprocate, are unique to khaini use behaviors in India. These items are also more appropriate for the Indian context than some items from the GN-STBQ^11^ and BQDS^15^ due to a lack of policies restricting the use of smokeless tobacco in public places^27^ and easy accessibility of tobacco shops^28^ in India. The KSLTDS also avoids the problems of using quantity/frequency items to assess physical dependence, which results from the variations in tobacco content, the quantity of a single dose, duration of use (dipping/chewing time) and nicotine yield noted for SLT products^9^. Rather, the KSLTDS scale included items assessing the experience of cravings after non-use for 30 minutes, increased intensity of cravings and waking at night to use khaini. The factors of KSLTDS were consistent with those constructed from previous SLT scales, supporting a high content validity of the newly developed measure. Despite the relatively small number of items, it can be claimed that the KSLTDS scale covered the important dimensions of dependence on khaini. These features ensured adequate levels of content validity and reduced respondent burden in completing the KSLTDS.

The result supports the theoretical framework by confirming that dependence on SLT is multidimensional, with various physical, psychological, behavioral and sociocultural factors responsible for dependence on khaini use^24^. The internal consistency of the KSLTDS sub-scales (0.87–0.90) was higher than some existing SLT scales where Cronbach’s alpha ranged from 0.30–0.69^6-9,11^. The assessment of criterion validity using cotinine showed a moderate correlation (ρ=0.43, p=0.0002) with total scores of the KSLTDS, which was higher than existing SLT scales GN-STBQ (r=0.02)^11^ and SSTDS (r=0.03)^11^. The cotinine levels were highly correlated with frequency of khaini use in the study, demonstrating a relationship between frequency of use and cotinine levels as established in earlier studies^12^. All three subscales and the total score of the KSLTDS were moderately correlated with cotinine level, indicating sound criterion validity. A moderate correlation between KSLTDS total scale and three subscales with the FTND-ST^7^ total score confirmed acceptable convergent validity. Similar to KSLTDS, existing scales OSSTD^12^ and TDS^13^ had moderate correlation with FTND-ST on assessing convergent validity.

The KSLTDS is more comprehensive than existing scales which focused on either the physical dependence (FTND-ST)^7^ or behavioral dependence (GNST-BQ)^11^. The KSLTDS included new items unique to the sociocultural context of dependence on SLT products in the South-Asia region which have not been included in existing SLT dependence scales which were developed primarily in Western or high-income countries. In addition, the KSLTDS development and evaluation process highlighted some important behaviors which relate to dependence (misconceptions responsible for use, social and family acceptance, environmental cues and lack of restrictions). Addressing these behaviors through behavior modification strategies and evidence-based counselling techniques is likely to help provide effective cessation treatment for khaini users. Further, the KSLTDS could be modified, adapted, and after validation, potentially be used with other highly prevalent smokeless tobacco products (e.g. gutkha) consumed in South Asia. Various factors responsible for dependence and continued khaini use need to be addressed through education and awareness campaigns to educate khaini users and develop policies regulating khaini use.

### Limitations

Our study has some limitations. First, the validation study was conducted in a tertiary care facility in one urban area and may not be representative of khaini users from rural areas or other socioeconomically different areas in India. As the scale was new, we did not conduct a confirmatory factor analysis of the KSLTDS. It is necessary to confirm the three-factor structure in another large sample of khaini users in future studies. Another limitation is that the predictive, test-retest and concurrent validity of the KSLTDS was not assessed, and future studies should consider examining these psychometric properties^12^.

## CONCLUSIONS

This study sought to address the need for valid and reliable scales to assess dependence on SLT. The KSLTDS is the first scale to be developed to measure dependence on khaini use in a manner appropriate to SLT users in India. The 20-item KSLTDS is relatively short yet comprehensive and was developed using a rigorous evidence-based process involving khaini users. The KSLTDS has acceptable psychometric properties and includes important aspects of dependence, making it a useful scale for both research and treatment. Further studies are required to re-validate the psychometric properties of the KSLTDS with other khaini user populations.

## Data Availability

The data supporting this research are available from the authors on reasonable request.
