# Erratum: Development and psychometric evaluation of the Khaini Smokeless Tobacco Dependence Scale

**DOI:** 10.18332/tid/167784

**Published:** 2023-06-14

**Authors:** European Publishing Production Team

**Affiliations:** 1 European Publishing, Heraklion, Greece

**Keywords:** dependence, psychometric properties, scale development, khaini, smokeless tobacco

**By Vaibhav P. Thawal, Christine Paul, Erin Nolan, Flora Tzelepis**

**Tobacco Induced Diseases, Volume 21, Issue March, Pages 1-14**

**Publish date: 15 March 2023**

**DOI: https://doi.org/10.18332/tid/160073**

In the original article, in the 13th page, the accessed date in reference 1 should have been changed to "Accessed February 10, 2022", in reference 3 to "Accessed May 20, 2022", in reference 4 to "Accessed April 15, 2022" whilst in page 14, the accessed date of the reference 25 should have been changed to "Accessed May 20, 2022" and of reference 26 to "Accessed May 20, 2022".

Furthermore "Acknowledgements" section should have been placed before the section "Conflicts of Interest".

Finally, the approval dates in the section "Ethical approval and informed consent" were missing and now the section has been corrected as follows: "Approval number: HREC-H-2018-0401; Date: 6 November 2018) and from the Joint Ethics Committee, Narotam Sekhsaria Foundation-Salaam Bombay Foundation, Mumbai, India (Approval number: JEC/NSF-SBF/2019/02; Date: 19 February 2019)". The mentioned changes are corrected also online.

