# Supplementary material for: Development and psychometric evaluation of the Khaini Smokeless Tobacco Dependence Scale
Source: Tob Induc Dis. 2023 Mar 15;21:40. doi: 10.18332/tid/160073 (PMC10016084; doi:10.18332/tid/160073)
Supplement: Supplementary file 1 [file TID-21-40-s1.pdf]

Supplementary information

List of domains used in the theoretical framework of dependence

Withdrawal; carving; tolerance; relapse, [1] cultural practices,[2] social norms,[2] activities associated with use[2] learned effects or misconceptions,[3] environmental factors,[3] cognitive associations,[4] family traditions,[4] positive enforcement,[5] negative enforcement,[5] habitual use,[5] addictive use,[5] appetite control,[6] situation specific use,[7] psychological factors (positive enforcement, negative enforcement, use to relax, use when alone, use when nervous or tense etc.),[5,6] automatic use,[8] sensorimotor manipulation,[8] use despite knowledge of health effects and persistent desire or effort to quit/ unsuccessful effort to cut down;[9] using in larger amounts or longer then intended; time spent in obtaining or using; recurrent tobacco use resulting in a failure to fulfil major role; use despite having persistent or recurrent social or interpersonal problems caused by the effects of tobacco; important social, occupational or recreational activities given up or reduced.[10]

NicAlert test strips:

The test strip displays seven zones with each zone representing a range of level of cotinine concentration in ng/ml. The results were recorded with seven levels from 0 to 6 with increasing cotinine concentration from 0 -10 ng/mL, 10-30, 30-100, 100-200, 200-500, 500-1000, >1000.[11]

Process followed for translation of the interview guide for in-depth interviews.

The interview guide was translated and back translated into Hindi and Marathi language through an external professional agency. The guides were further rechecked by the first author who speaks English, Hindi and Marathi.

Process followed for translation of the draft KSLTDS scale.

The scale items were initially developed in English, with regular reference to the language and terms used by study participants. All the scale items were translated in Hindi and Marathi language with reference to the terms used by participants during phase 1. The translations were done by two linguistic experts having good proficiency in Hindi and Marathi language. The translated version was then rechecked by the first author and a few linguistic corrections were made.

Supplementary table 1: Item-total correlation for draft KSLTDS, India, 2019-2020, Phase 3 scale evaluation (Step 4: Survey administration), N=323

**Items**	**Correlation ρ**
1. Khaini use per day	0.31
2. Minutes khaini on mouth	0.19
3. Minutes after awakening that first use khaini	-0.34
4. I experience cravings if I don’t use for 30 minutes	0.59
5. My cravings get stronger if I don’t use	0.73
6. I find myself using khaini routinely without cravings	0.73
7. Few things give me pleasure each day like using khaini	-0.00
8. I use khaini every time I go to the washroom	0.48
9. My khaini use is a routine habit similar to eating	0.78
10. I use khaini when I have to focus on a task	0.54
11. I sometimes wake up at night to use khaini	0.65
12. I feel bored if I don’t use khaini for some time	0.29
13. If I don’t use khaini I feel I am missing an important daily activity	0.77
14. I use khaini more when I am sad tense stressed or worried	0.73
15. I feel energetic and fresh after using khaini	0.20
16. I feel anxious when I don’t have khaini available with me	0.69
17. I use khaini to relax myself	0.38
18. I have gradually increased the amount of khaini I use	0.69
19. I increase the amount of khaini I use to get a pleasant effect	0.75
20. I use khaini after meals or tea	0.38
21. I feel like using khaini if I see someone preparing or using it	0.17
22. I get drowsy if I don’t use khaini for some time	0.81
23. I would continue using khaini even if I have cancer sores, mouth ulcers or loose teeth	0.67
24. I use khaini even if I am so ill that I am in bed most of the day	0.65
25. It would be difficult for me to quit my khaini use completely	0.80
26. I ask people I don’t know to share their khaini with me	0.55
27. I am aware of the quantity of khaini and plan to buy more so I won’t run out	0.68
28. I use khaini in front of my friends	0.79
29. I use khaini in front of my family	0.63
30. I will share khaini with others so they will share with me when I need it	0.58
31. I do not share khaini with other khaini users	-0.64
32. Most people I spend time with are khaini users	0.46

ρ: correlation coefficient

1. Piper ME, McCarthy DE, Baker TB. Assessing tobacco dependence: a guide to measure evaluation and selection. *Nicotine & Tobacco Research*. 2006;8(3):339-351.
2. Quintero G, Nichter M. The semantics of addiction: moving beyond expert models to lay understandings. *Journal of Psychoactive Drugs*. 1996;28(3):219-228.
3. Bandura A. Social foundation of thought and action. Englewood cliffs, NJ: prentice Hall; 1986.
4. Marlatt GA. Relapse prevention: Theoretical rationale and overview of the model. *Relapse prevention*. 1985:3-70.
5. Tomkins SS. Psychological model for smoking behavior. *American Journal of Public Health and the Nations Health*. 1966;56(12_Suppl):17-20.
6. Russell M, Peto J, Patel U. The classification of smoking by factorial structure of motives. *Journal of the Royal Statistical Society: Series A (General)*. 1974;137(3):313-333.
7. Best JA, Hakstian AR. A situation-specific model for smoking behavior. *The Psychology of Social Situations*. Elsevier; 1981:454-473.
8. Tate JC, Pomerleau CS, Pomerleau OF. Pharmacological and non-pharmacological smoking motives: a replication and extension. *Addiction*. 1994;89(3):321-330.
9. Baker TB, Breslau N, Covey L, Shiffman S. DSM criteria for tobacco use disorder and tobacco withdrawal: A critique and proposed revisions for DSM-5. *Addiction*. 2012;107(2):263-275.
10. Association AP. *Diagnostic and statistical manual of mental disorders.* 5th ed ed. 2013.
11. Asha V, Dhanya M. Immunochromatographic Assessment of Salivary Cotinine and Its Correlation With Nicotine Dependence in Tobacco Chewers. *J*. Jun 2015;20(2):159-63. doi:https://dx.doi.org/10.15430/JCP.2015.20.2.159

STROBE Statement—Checklist of items that should be included in reports of ***cross-sectional studies***

	Item No	Recommendation	Page No
**Title and abstract**	1	(*a*) Indicate the study's design with a commonly used term in the title or the abstract	1
		(*b*) Provide in the abstract an informative and balanced summary of what was done and what was found	Attached separately
**Introduction**			
Background/rationale	2	Explain the scientific background and rationale for the investigation being reported	1 and 2
Objectives	3	State specific objectives, including any prespecified hypotheses	3
**Methods**			
Study design	4	Present key elements of study design early in the paper	3
Setting	5	Describe the setting, locations, and relevant dates, including periods of recruitment, exposure, follow-up, and data collection	3
Participants	6	(*a*) Give the eligibility criteria, and the sources and methods of selection of participants	5 (and Annexure-A)
Variables	7	Clearly define all outcomes, exposures, predictors, potential confounders, and effect modifiers. Give diagnostic criteria, if applicable	NA
Data sources/ measurement	8*	For each variable of interest, give sources of data and details of methods of assessment (measurement). Describe comparability of assessment methods if there is more than one group	3 to 6
Bias	9	Describe any efforts to address potential sources of bias	NA
Study size	10	Explain how the study size was arrived at	5
Quantitative variables	11	Explain how quantitative variables were handled in the analyses. If applicable, describe which groupings were chosen and why	5 and 6
Statistical methods	12	(*a*) Describe all statistical methods, including those used to control for confounding	5 and 6
		(*b*) Describe any methods used to examine subgroups and interactions	NA
		(*c*) Explain how missing data were addressed	NA
		(*d*) If applicable, describe analytical methods taking account of sampling strategy	NA
		(*e*) Describe any sensitivity analyses	NA

**Results**			
Participants	13*	(a) Report numbers of individuals at each stage of study—eg numbers potentially eligible, examined for eligibility, confirmed eligible, included in the study, completing follow-up, and analysed	Figure 1
		(b) Give reasons for non-participation at each stage	Figure 2
		(c) Consider use of a flow diagram	Figure 2
Descriptive data	14*	(a) Give characteristics of study participants (eg demographic, clinical, social) and information on exposures and potential confounders	Table 1
		(b) Indicate number of participants with missing data for each variable of interest	NA
Outcome data	15*	Report numbers of outcome events or summary measures	Table 4
Main results	16	(*a*) Give unadjusted estimates and, if applicable, confounder-adjusted estimates and their precision (eg, 95% confidence interval). Make clear which confounders were adjusted for and why they were included	NA
		(*b*) Report category boundaries when continuous variables were categorized	NA
		(*c*) If relevant, consider translating estimates of relative risk into absolute risk for a meaningful time period	NA
Other analyses	17	Report other analyses done—eg analyses of subgroups and interactions, and sensitivity analyses	NA
**Discussion**			
Key results	18	Summarise key results with reference to study objectives	6 and 7
Limitations	19	Discuss limitations of the study, taking into account sources of potential bias or imprecision. Discuss both direction and magnitude of any potential bias	8 and 9
Interpretation	20	Give a cautious overall interpretation of results considering objectives, limitations, multiplicity of analyses, results from similar studies, and other relevant evidence	8 and 9
Generalisability	21	Discuss the generalisability (external validity) of the study results	8 and 9
**Other information**			
Funding	22	Give the source of funding and the role of the funders for the present study and, if applicable, for the original study on which the present article is based	Information submitted separately

© 2023 Twawal V.P. et al.
